# Diversity of *Pneumocystis jirovecii* Across Europe: A Multicentre Observational Study

**DOI:** 10.1016/j.ebiom.2017.06.027

**Published:** 2017-06-29

**Authors:** Alexandre Alanio, Maud Gits-Muselli, Nicolas Guigue, Marie Desnos-Ollivier, Enrique J. Calderon, David Di Cave, Damien Dupont, Axel Hamprecht, Philippe M. Hauser, Jannik Helweg-Larsen, Marta Kicia, Katrien Lagrou, Martina Lengerova, Olga Matos, Willem J.G. Melchers, Florent Morio, Gilles Nevez, Anne Totet, Lewis P. White, Stéphane Bretagne

**Affiliations:** a Laboratoire de Parasitologie-Mycologie, AP-HP, Groupe Hospitalier Saint-Louis-Lariboisière-Fernand-Widal, Paris, France; b Université Paris Diderot, Sorbonne Paris Cité, Paris, France; c Institut Pasteur, CNRS, Unité de Mycologie Moléculaire, Centre National de Référence Mycoses Invasives et Antifongiques, URA3012, Paris, France; d CIBER de Epidemiología y Salud Pública, Instituto de Biomedicina de Sevilla, Hospital Universitario Virgen del Rocío, CSIC, Universidad de Sevilla, Spain; e Department of Clinical Sciences and Translational Medicine, University of Rome “Tor Vergata”, Italy; f Hospices Civils de Lyon, Institut des Agents Infectieux, Parasitologie Mycologie, Hôpital de la Croix-Rousse, Integrative Physiology of the Brain Arousal Systems, Centre de Recherche en Neurosciences de Lyon, INSERM U1028-CNRS UMR 5292, Faculté de Médecine, Université Claude Bernard Lyon 1, Lyon F-69000, France; g Institute for Medical Microbiology, Immunology and Hygiene, University Hospital Cologne, Germany; h Institute of Microbiology, Lausanne University Hospital, University of Lausanne, Lausanne, Switzerland; i Department of Infectious Diseases, Rigshospitalet-Copenhagen University Hospital, Copenhagen, Denmark; j Department of Biology & Medical Parasitology, Wroclaw Medical University, Wroclaw, Poland; k Department of Microbiology and Immunology, Catholic University Leuven, Leuven, Belgium and National Reference Centre for Mycosis, Department of Laboratory Medicine, University Hospitals Leuven, Leuven, Belgium; l Department of Internal Medicine - Hematology and Oncology, University Hospital Brno, Brno, Czech Republic; m TB, HIV and Opportunistic Diseases and Pathogens, Global Health and Tropical Medicine, Lisboa, Portugal; n Instituto de Higiene e Medicina Tropical, Universidade NOVA de Lisboa, Lisboa, Portugal; o Department of medical microbiology, Radboud University Medical Centre, Nijmegen, The Netherlands; p Parasitology and Mycology laboratory, Nantes University Hospital, Nantes, France; q University of Brest, GEIHP EA 3142, Laboratory of Parasitology and Mycology, Brest University Hospital, Brest, France; r University of Picardy-Jules Verne, EA 4285 UMR-I 01 INERIS, Department of Parasitology and Mycology, Amiens University Hospital, Amiens, France; s Public Health Wales, Microbiology Cardiff, UHW, Heath Park, Cardiff, UK

**Keywords:** Pneumocystis jirovecii, Genotyping, Europe, Transmission, Mixed infection, MLS typing, Microsatellites

## Abstract

*Pneumocystis jirovecii* is an airborne human-specific ascomycetous fungus responsible for *Pneumocystis* pneumonia (PCP) in immunocompromised patients, affecting > 500,000 patients per year (www.gaffi.org). The understanding of its epidemiology is limited by the lack of standardised culture. Recent genotyping data suggests a limited genetic diversity of *P. jirovecii*. The objective of the study was to assess the diversity of *P. jirovecii* across European hospitals and analyse *P. jirovecii* diversity in respect to clinical data obtained from the patients.

Genotyping was performed using six already validated short tandem repeat (STR) markers on 249 samples (median: 17 per centre interquartile range [11 − 20]) from PCP patients of 16 European centres.

Mixtures of STR markers (i.e., ≥ 2 alleles for ≥ 1 locus) were detected in 67.6% (interquartile range [61.4; 76.5]) of the samples. Mixture was significantly associated with the underlying disease of the patient, with an increased proportion in HIV patients (78.3%) and a decreased proportion in renal transplant recipients (33.3%) (p < 0.001). The distribution of the alleles was significantly different (p < 0.001) according to the centres in three out of six markers. In analysable samples, 201 combinations were observed corresponding to 137 genotypes: 116 genotypes were country-specific; 12 in two; six in three; and two in four and one in five countries. Nine genotypes were recorded more than once in a given country. Genotype 123 (Gt123) was significantly associated with France (14/15, p < 0.001) and Gt16 with Belgium (5/5, p < 0.001). More specifically, Gt123 was observed mainly in France (14/15/16 patients) and in renal transplant patient (13/15).

Our study showed the wide population diversity across Europe, with evidence of local clusters of patients harbouring a given genotype. These data suggest a specific association between genotype and underlying disease, with evidence of a different natural history of PCP in HIV patients and renal transplant recipients.

## Highlights

• Limited number of *P. jirovecii* genetic complexes across Europe but high number of genotypes uncovered.
• Association between mixture of genotype and HIV patients.
• Specific relationship between renal transplant recipients and specific *P. jirovecii* genotypes with the description of multiple local transmissions.
• Potential geographical and temporal dissemination of a given genotype within an area of 600 km.

*Pneumocystis jirovecii* is an airborne opportunistic fungal organism. As it is not culturable, genotyping and population genetics is difficult. We implemented recently a method allowing us to genotype the largest cohort of *P. jirovecii* isolates (n = 249) across Europe. We evidenced that HIV patients were more prone to harbour mixtures of genotypes compared to other underlying diseases, that local transmission of a given genotype was frequent in renal transplant recipients with potential dissemination within a timescale of several years and an area of 600 km. This work advances our understanding of the population structure and the transmission of *P. jirovecii* in humans.

## 1 Introduction

*Pneumocystis jirovecii* is a life-threatening atypical fungal organism for which our knowledge of its epidemiology and basic biology is limited, largely due to an inability to reproducibly culture the microorganism in vitro (Thomas and Limper, 2007). *Pneumocystis* species has been coevolving with each mammal species for 100 millions years leading to the specificity of *P. jirovecii* to humans (Aliouat-Denis et al., 2008). Individuals are exposed to *P. jirovecii* in childhood, with > 80% of children being seropositive before the age of 2 years (Bishop and Kovacs, 2003, Meuwissen et al., 1977, Peglow et al., 1990, Pifer et al., 1978, Vargas et al., 2001). This organism is therefore considered as a pulmonary resident of immunocompetent individuals who carry *P. jirovecii,* at least transiently, in their respiratory tract (Gigliotti and Wright, 2012). *P. jirovecii* can then circulate constantly between normal hosts as suggested by the high proportion of mixed infections (Alanio and Bretagne, 2017).

Genotyping systems have been developed in the last 20 years to explore the pathophysiology of the infection (Pneumocystis pneumonia, PCP) and to trace the transmission of *P. jirovecii* in specific areas such as hospital wards. PCR single strand conformation polymorphism (PCR-SSCP) of nuclear and mitochondrial loci allowed the analysis of several isolates associated with clustered infections (Hauser et al., 1997a, Hauser et al., 1997b, Nahimana et al., 2000). Sequencing of internal transcribed spacers (ITS), which locus is unique in the nuclear genome (Helweg-Larsen et al., 2001, Le Gal et al., 2012, Tsolaki et al., 1996), and sequencing of mitochondrial large subunit ribosomal loci have also been used (Keely et al., 1995). A multi-locus sequence typing (MLST) approach has also been implemented (Keely and Stringer, 1996) and a MLST scheme was optimized based on the sequencing of eight nuclear and mitochondrial loci (Maitte et al., 2013). The number of different genotypes described in literature was 43 using the PCR-SSCP method (Hauser, 2004), although > 60 genotypes were identified using ITS sequencing methods (Lu and Lee, 2008). Single nucleotide polymorphism (SNP) was also studied using single nucleotide primer extension in mitochondrial and genomic genes (Alanio et al., 2015, Esteves et al., 2011) and more recently, using ultra-deep-pyrosequencing (UDPS) to study three mitochondrial SNPs (Alanio et al., 2016).

An important feature of genotyping methods is the sensitivity when detecting minority alleles, since for *P. jirovecii* genotyping is performed directly on clinical samples because of the absence of culture. Although mixtures of two or more genotypes in one clinical sample have been reported, the rate of mixed infection varied from 5 to 25% using classical Sanger DNA sequencing (Esteves et al., 2010, Esteves et al., 2008, Helweg-Larsen et al., 2001) and single nucleotide primer extension, respectively (Alanio et al., 2015, Esteves et al., 2011), to 70% by both PCR-SSCP (Hauser et al., 2001) and Microsatellite Length Polymorphism (MLP) (Gits-Muselli et al., 2015, Parobek et al., 2014), and to > 90% of the samples using UDPS (Alanio et al., 2016).

To avoid sequencing and to test genetic variation differently to SNPs, MLP schemes have also been recently developed, targeting Short Tandem Repeat (STR) markers (Gits-Muselli et al., 2015, Parobek et al., 2014) identified once the *P. jirovecii* genome was available (Cissé et al., 2012). The first MLP genotyping method developed by Parobek et al., based on eight genetically unlinked markers, was used to genotype 63 clinical isolates from Africa (Uganda), Europe (Spain), and USA (San Francisco) (Parobek et al., 2014). A different MLP genotyping method based on six other STR markers was more recently developed (Gits-Muselli et al., 2015). This last MLP typing method is reproducible, cheap, with a high throughput and a high discriminatory power (DP) of 0.992 making it interesting to investigate transmission in hospital settings (Gits-Muselli et al., 2015, Robin et al., 2017).

The objective was to understand the diversity of this organism across Europe and to get insight into the geographical and temporal evolution of the different genotypes. The diversity of *P. jirovecii* isolates responsible for PCP across different European countries (n = 12) was investigated, in order to detect the most prevalent genotypes and to detect possible transmission within or between centres (n = 16) using this novel MLP typing method (Gits-Muselli et al., 2015).

## 2 Material and Methods

### 2.1 Patients and Clinical Samples Collection

**Fig. 1:**
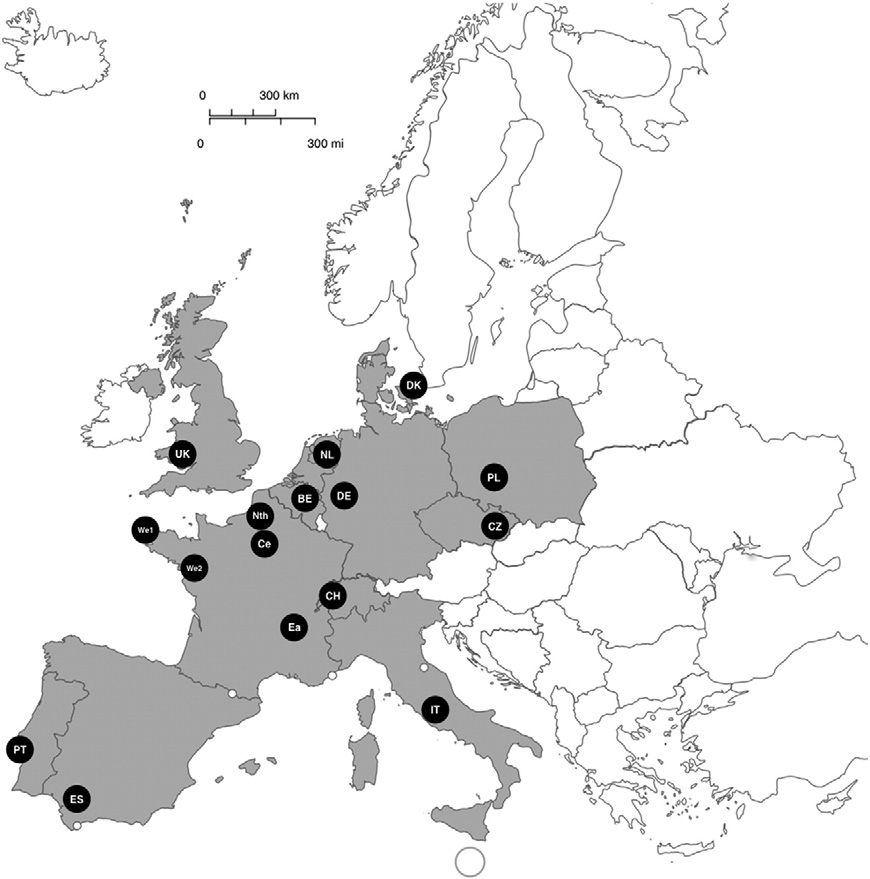
Distribution of centres (spot) and countries (grey) who participated to this study. The abbreviation of the centre is indicated within each spot.

Sixteen European centres were asked to send 25 frozen DNA samples obtained from respiratory samples from patients with PCP over the minimum period of time. These 16 centres across Europe included five French centres (Amiens University Hospital [Nth-FR]; Brest University Hospital, Brest [We1-FR], Nantes University Hospital, Nantes [We2-FR]; Hôpital de la Croix-Rousse, Lyon [Ea-FR] and Saint-Louis University Hospital, Paris [Ce-FR]), and one centre per country for Belgium [BE] (University Hospitals Leuven), Czech Republic [CZ] (University Hospital Brno), Denmark [DK] (Rigshospitalet-Copenhagen University Hospital), Germany [DE] (University Hospital Cologne), Italy [IT] (University of Roma “Tor Vergata”), Poland [PL] (Wroclaw Medical University), Portugal [PT] (Instituto de Higiene e Medicina Tropical, Universidade NOVA de Lisboa), Spain [ES] (Hospital Universitario Virgen del Rocío, Sevilla), Switzerland [CH] (Lausanne University Hospital), The Netherlands [NL] (Radboud University Medical Centre, Nijmegen), and United Kingdom [UK] (Public Health Wales, Microbiology Cardiff) (Fig. 1).

The samples consisted of DNAs extracted at the time of PCP diagnosis in the corresponding centre following local DNA extraction and storage procedure except for DK and ES who send frozen respiratory specimens which DNA was extracted in Ce-FR as already reported (Gits-Muselli et al., 2015). The samples were bronchoalveolar lavage (BAL) fluids (85.1%) and sputa (14.9%). The microscopy and local PCR results, including the quantification cycle (Cq) when quantitative PCR (qPCR) assays had been used were recorded. Data regarding date of PCP diagnosis, demographics, country of birth, and underlying disease or condition (HIV-infection, haematological malignancy, solid organ transplant, solid cancer, or immunological disorders) were collected. Only five paediatric cases were included.

### 2.2 STR Genotyping

The six STRs markers (#022, #108, #138, #189, #278, and #279) were amplified separately as previously described (Gits-Muselli et al., 2015). Briefly, the forward primers were tagged with FAM, HEX or ATTO565 fluorophores. PCR reactions were performed on a GeneAmp PCR System 9700 Thermocycler (Applied Biosystems) in a final volume of 20 μL. The reaction mixture was composed of 1 × AmpliTaq Gold buffer (Life technologies) with 0.25 μM of each primer, 2.5 mM of MgCl2, 0.8 μM of dNTPs, 0.25 UI of AmpliTaq Gold polymerase (Life technologies) and 2 μL of DNA. The PCR protocol was 10 min at 95°, followed by 35 cycles of 30 s at 95 °C (denaturation), 30 s at 56° (primer annealing) and 60 s at 72 °C (extension) followed by a final extension of 10 min at 72 °C. A calibration sample was used in each PCR run as an internal control. Then, 2 μL of the PCR product was prepared for fragment analysis by the addition of 18 μL of formamide (Life Technologies) and 1 μL of Genescan-500 LYZ Size Standard (Life Technologies). The lengths of the PCR fragments were determined by capillary electrophoresis (50 mm) on an ABI 3500 genetic analyser using denaturing polymer POP-7 (Life Technologies) at 60 °C. Analysis was performed with the ABI Gene mapper v4.1 software (Life Technologies).

### 2.3 Genotyping Analysis

The sample was included in the study if all six markers were successfully amplified and analysed. A unique combination of all six markers defined a genotype (Gt) or a strain, that was called fungal individual (FI), since culture is not possible in *P. jirovecii*. Gt could be delineated if (i) each of the six STR markers were pure (no additional peak corresponding to a smaller or larger amplicon) or (ii) only one marker had multiple amplicons (one to three additional detectable peaks corresponding to a smaller or larger amplicon). In the latter case, the interpretation was mixture of two or three FIs from genotypes different in only one marker. When several alleles were observed for > 2 loci, Gt and FIs could not be individualized and delineated. Relatedness between genotypes was investigated by comparing allelic profiles with the minimum spanning tree (MStree) method (BioNumerics software v6.5, Applied Maths Inc., Austin, TX) as previously described (Gits-Muselli et al., 2015). Briefly, alleles were treated as multistate categories based on an infinite allele model (i.e., all changes are equally likely). Genetic clusters were defined as group of genotypes that have a single allelic mismatch with at least one other member of the group. Singletons were defined as genotypes that were not grouped into clonal complexes (at least two allelic mismatches with other genotype). The number of repeat differences between ancestral and derived alleles was computed for each link of one mismatch along the Minimum Spanning Tree. The classical criterion of one allelic mismatch to group genotypes in clonal complexes was used (Feil, 2004). The discriminatory power (DP) as an indicator of the genetic diversity was calculated with Simpson's diversity index, as described previously (Hunter and Gaston, 1988).

### 2.4 Statistical and Graph Analysis

Chi-2 test and Fisher's exact test were used for contingency tables analyses for calculation of statistical association between genotypes and mixtures, genotypes and clinical data, mixture and clinical data, genotypes and centres mixture and centres. Median and interquartile ranges are described in the text. *P* values of < 0.05 were considered significant. Graphs (grouped columns of the alleles recorded in the different centres) and statistical analyses were performed by using Prism 6.0 (GraphPad Software Inc., La Jolla, CA, USA).

### 2.5 Ethics Statement

This study was a non-interventional study with no change in the usual procedures. Biological material and clinical data were obtained only for standard diagnostic following physicians' prescriptions with no specific sampling. For France, according to the French Health Public Law (CSP Art L1121-1.1), such protocol does not require approval of an ethics committee and is exempted from specific informed consent application. However, all patients were informed and signed documents for non-opposition to this study. Data file was declared and approved to the French data protection agency (no. 1818924). In specific countries, approval from local ethic committee was obtained when required.

## 3 Results

**Fig. 2:**
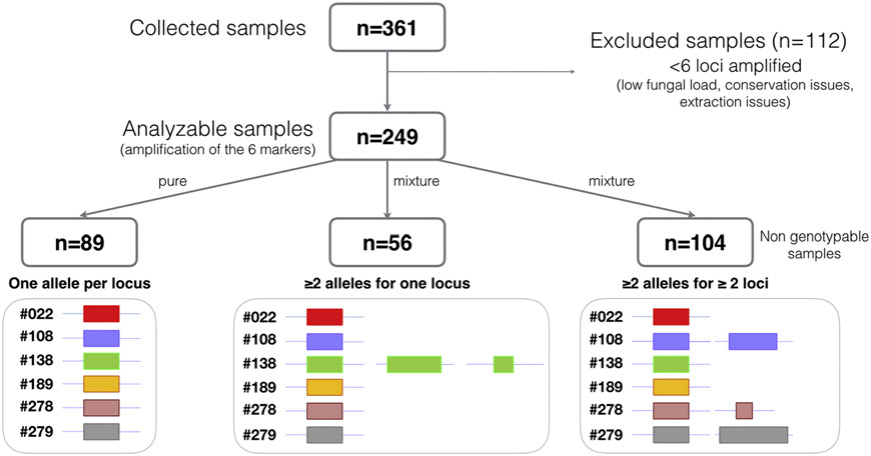
Flow chart representing the number of samples analysed and classified in this study. The number of isolates harbouring pure (one allele per locus), genotypable mixtures (≥ 2 alleles for one locus) and non-genotypable mixtures (≥ 2 alleles for ≥ 2 loci).

A total of 361 samples from 361 patients with a median of 25 samples per centre [19–25], obtained from 1998 to 2015 (97.2% collected after 2009), were sent to Saint-Louis Hospital, Paris, France for genotyping. All samples had microscopic evidence of the presence of asci or trophic forms using standard staining or anti-*Pneumocystis* immunofluorescence, or were PCR-positive with high fungal load according to the local qPCR assay. Of these 361 DNA samples, the six STR markers were correctly amplified in 249 (69.0%) samples from 249 patients (Fig. 2). Samples with amplification failure of one or more of the 6 markers (112/361; 31%) were further excluded. The median number of successfully amplified samples per centre was 17 (11 − 20). The qPCR assays performed locally on these samples were heterogeneous (mainly in-house PCR targeting either mtLSU, Kex, or MSG genes, including already published assays (Alanio et al., 2011, Larsen et al., 2002, Totet et al., 2003). The median Cq was 25 [22–28]. Male/female ratio was 1.83 and the median age was 55 years. The patients' predisposing diseases or conditions were AIDS (36.9%), haematological malignancy (19.7%), renal transplantation (13.3%), and other causes of immunosuppression (25.3%), with this piece of information not available for 4.8% of cases.

### 3.1 Mixtures of Genotypes

Genotyping analysis revealed that 160 out of 249 (64.2%) samples contained mixtures of genotypes including 56 samples harbouring multiple alleles at one locus and 104 samples harbouring multiple alleles at ≥ 2 loci (Fig. 2). Of note, 61.3% of the 31 excluded samples because of one locus amplification failure also contained mixed genotypes. The median percentage of mixtures per centre was 67.5%, interquartile range [61.4; 76.5] with a trend toward a different proportion of mixture in the different centres (Chi2 test; p = 0.090). The mixtures were significantly associated with the underlying disease (p < 0.001), with an increased proportion of mixture in HIV-positive patients (78.3%) compared to HIV-negative patients (55.9%, p < 0.001) and a decreased proportion of mixtures in renal transplant recipients (33.3%) compared to other patients (69.6%, p < 0.001).

### 3.2 Genotyping Analysis

**Fig. 3:**
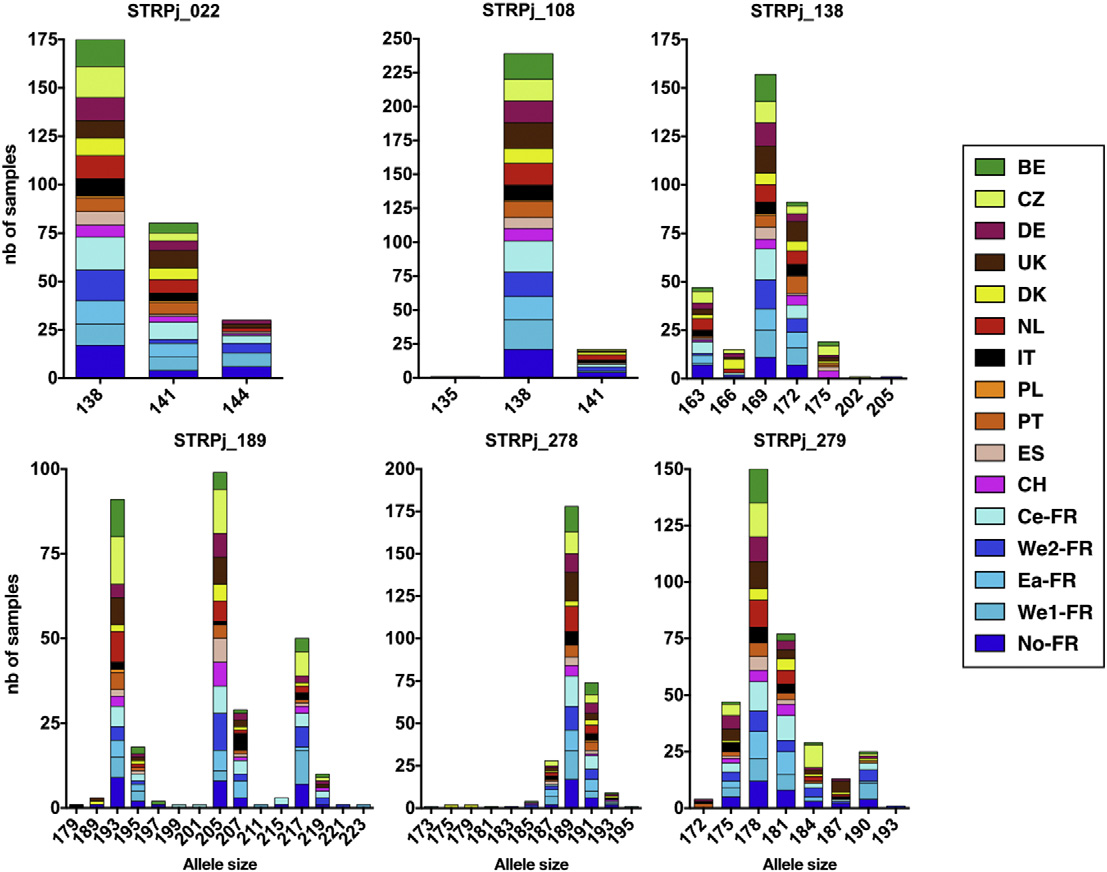
Allele distribution for the 249 samples from the 16 European centres. The number of isolates harbouring a specific allele size was collected per centre and pooled as a function of the observed allele sizes for each marker. Coloured boxes allowed identification of the centre.

Based on the analysis of the 249 samples, 3 distinct alleles were observed for STR #022, 3 for #108, 7 for #138, 15 for #189, 11 for #278, and 8 for #279. The distribution of the alleles at each centre is shown in Fig. 3. The distribution of alleles was significantly different according to the centre for STR #138 (p = 0.011), #278 (p = 0.006), and #279 (p < 0.001).

**Fig. 4:**
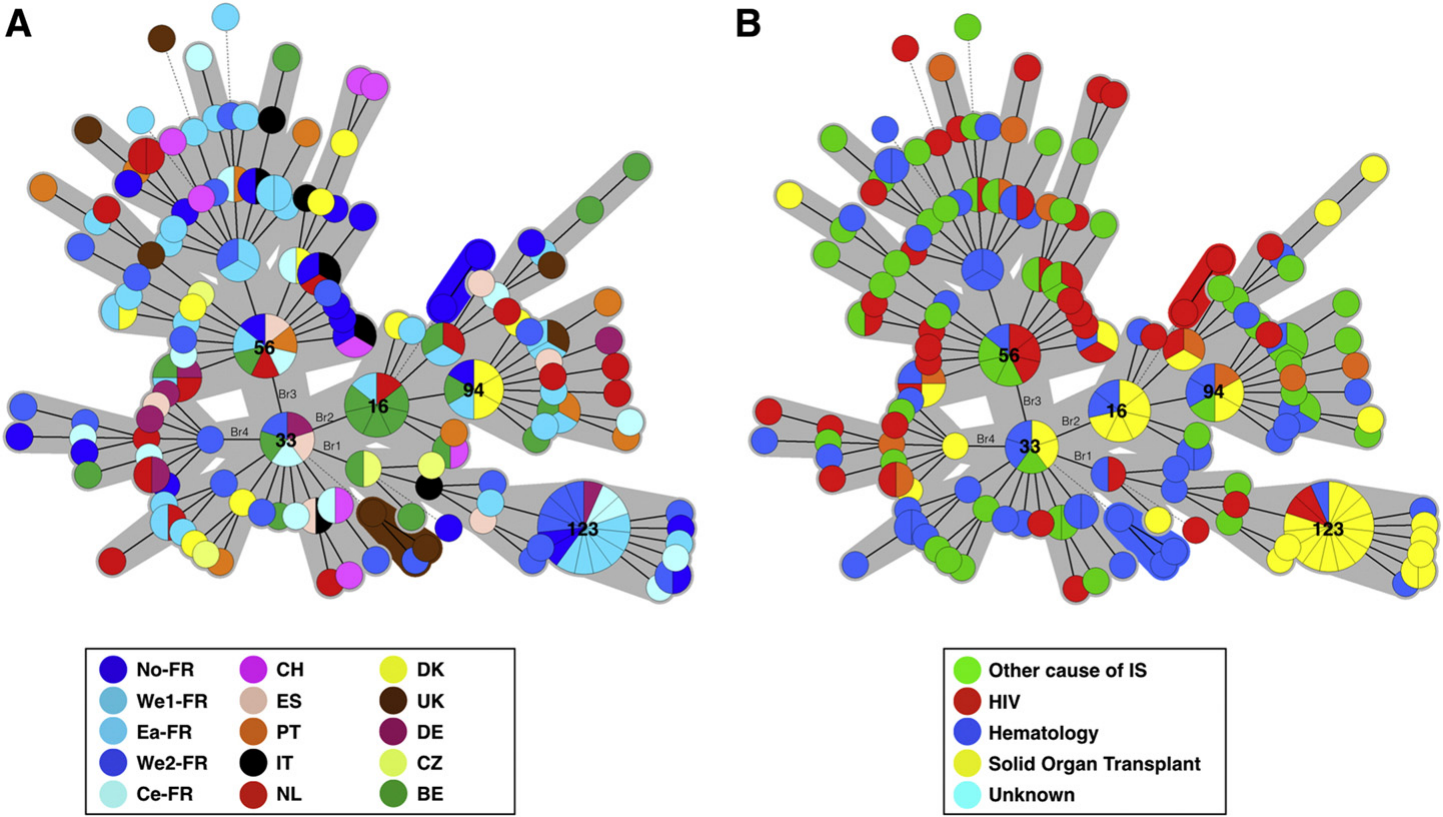
Minimum spanning tree analysis of 137 genotypes from 145 samples (201 fungal individuals) harbouring a unique genotype (one allele per marker) or multiple genotypes (multiple alleles in one marker). The number of allelic mismatches among STR profiles was used as distance. Each circle corresponds to one genotype (Gt), with its arbitrary number indicated next to it. The size of the circle is correlated with the number of isolates possessing the corresponding genotype, from one (smallest circle) to thirteen (Gt123). Dark, dashed and thin connecting bars correspond to one, 2 or > 2 different markers observed between linked genotypes. Coloured zones surrounding some groups of circles indicate that these profiles belong to the same genetic cluster, meaning that they have a single allelic mismatch with at least one other member of the group. Cluster 2, which was significantly associated with renal transplant recipients, is shown by a dashed line. The colour of the circles indicates the underlying disease of the patient in whom this specific genotype was recovered (Green, HIV patient; Red, haematology patient; Purple, renal transplant recipient; Yellow, other cause of immunosuppression). (For interpretation of the references to colour in this figure legend, the reader is referred to the web version of this article.)

Genotype assignment was performed for 145 samples harbouring one allele for each locus (n = 89) or 2–3 alleles for one locus only (n = 56). To ascribe the different peaks to a given genotype was not possible in samples harbouring mixtures at more than one marker (n = 104). Therefore, genotype distribution analysis was performed on 145 samples out of the initial 249 samples (58%) and revealed 137 genotypes from 201 FIs (Fig. 4). MST analysis showed a repartition in one major complex (n = 128 genotypes), two minor clonal complexes (2 and 3 genotypes, respectively), and singletons (n = 4 genotypes) (Fig. 4A). Gt33 (found in Ce-FR, We2-FR, BE, ES and DE) was the founder genotype from which all genotypes derived with 11 genetic branches (Br1 to Br11) generated from it. From these 11 branches, four main branches (Br1 to Br4) can be delineated (Fig. 4A). Br1 was composed of 16 genotypes (31 FIs), Br2 of 32 genotypes (50 FIs), Br3 of 50 genotypes (70 FIs), and Br4 of 14 genotypes (15 FIs). The proportion of solid organ transplant patients significantly increased in Br1 compared to the other underlying diseases (42.1% vs. 6.8%; p < 0.001) and significantly decreased in Br3 compared to the other underlying diseases (4.8% vs. 15.5%; p = 0.044) (Fig. 4B). The repartition of the samples regarding the country of origin of the patients and date of recovery did not show any clustering (data not shown).

The DP was 0.991 based on the analysis of 201 FIs. Since highly independent isolates must be used for DP calculation (Hunter and Gaston, 1988), renal transplant recipients harbouring the same genotype were excluded because direct transmission between patients could be suspected. Thus, 12 FIs from three centres were excluded from this analysis (Gt123 [-7]; Gt16 [-4]; Gt94 [− 2]) and the final DP was 0.995.

### 3.3 Geographical Distribution of the Genotypes

Of the 137 genotypes, 116 (84.7%) were individual to a single country, 12 (8.8%) in two, six (4.4%) in three, two (1.5%) in 4 and one (0.7%) in five countries (Table 1, Supplementary Table 1, Supplementary Table 2). Of note, one genotype (Gt56) was found in seven different centres (five countries). The DP varied from centre to centre with a median at 1.00 (range [0,93–1.00]. Eight centres (Ea-FR, CH, ES, PT, IT, DK, DE, CZ) had a DP at 100% and three centres had a DP < 0.96 (We1-FR [0.931], UK [0.955] and BE [0.947]).

**Table 1:** The number of different genotypes observed across countries and centres.

	Number of countries where identical genotypes were recovered						
	1	2	3	4	5	6	7
Nb of genotypes (n = 137)	116	12	6	2	1	0	0

	Number of centres where identical genotypes were recovered						
	1	2	3	4	5	6	7

Nb of genotypes (n = 137)	113	13	6	2	2	0	1

Ten genotypes were found more than once in a given country. Notably, Gt94 was found in 4/19 (15.8%) patients in UK, Gt16 was found in 6/19 (31.6%) patients in Belgium, and Gt123 was found in 15/108 (13.9%) patients in France. Of note, Gt94 and Gt16 were found in three countries respectively (four centres for Gt94) and Gt123 in two countries (five centres).

Two out of the four patients harbouring Gt94 were renal transplant recipients representing all the renal transplant recipients of the cohort (Table 2). Five out of the six patients with Gt16 were renal transplant recipients and Gt16 was found in 5/9 (55.6%) renal transplant recipients tested. Gt123 was found in 15 patients including 14 from west, north and centre of France (six from centre We1-FR, 5 from centre We2-FR and 2 from No-FR and 2 from Ce-FR). No Gt123 was observed in Ea-FR. Among the 15 French patients harbouring Gt123, 13 (86.7%) were from renal transplant patients sampled from 2009 to 2015. In the centres with presence of Gt123, a median of 66.7% (interquartile range: 54.2–91.7) of the renal transplant recipients and 16.2% (interquartile: range 8.4–26.3) of the other patients tested harboured the Gt123. Indeed, an increased representation of Gt123 was observed in SOT patients compared to other underlying diseases (*p* < 0.001).

**Table 2:** Characteristics of the patients and samples harbouring Gt16, Gt94 and Gt123 in their respective country.

Sample/patient n°	Centre	Type of infection	Date of recovery	Sample type	Gender	Age	Underlying disease	Country of origin of the patient	Episodes	Prophylaxis	Genotype
4	Nth-FR	Pure	19/08/13	BAL	F	65	Renal transplant	France	First	No	123
18	Nth-FR	Pure	23/01/15	BAL	M	58	Renal transplant	France	First	No	123
28	We1-FR	Pure	28/05/09	BAL	M	55	Renal transplant	France	First	no	123
31	We1-FR	Pure	26/08/10	BAL	F	56	Renal transplant	France	First	no	123
33	We1-FR	Pure	27/09/10	BAL	M	45	Renal transplant	France	First	no	123
34	We1-FR	Pure	09/09/11	BAL	M	55	Renal transplant	France	First	na	123
40	We1-FR	Pure	25/11/10	BAL	M	55	Renal transplant	France	First	no	123
43	We1-FR	Pure	13/11/09	BAL	M	26	Haematology	France	First	yes	123
156	We2-FR	Pure	10/10/13	BAL	M	46	Renal transplant	France	First	No	123
164	We2-FR	Pure	19/07/14	BAL	M	69	Other cause of immunosupression	France	First	No	123
171	We2-FR	Mixed	02/03/15	BAL	F	26	Renal transplant	France	First	na	123
175	We2-FR	Pure	26/03/12	BA	M	32	Renal transplant	France	First	No	123
176	We2-FR	Pure	08/06/12	BAL	M	60	Renal transplant	France	First	No	123
180	Ce-FR	Pure	23/04/15	Sputum	F	67	Renal transplant	Algeria	First	No	123
183	Ce-FR	Pure	17/10/14	BAL	M	68	Renal transplant	Caribbean island	First	No	123
46	UK	Pure	02/03/15	BAL	F	62	na	UK	na	yes	94
49	UK	Mixed	01/09/14	BAL	M	52	HIV-infected	UK	na	yes	94
52	UK	Pure	13/03/14	BAL	M	62	Renal transplant	UK	na	na	94
59	UK	Pure	17/03/15	BAL	M	71	Renal transplant	UK	na	na	94
231	BE	Pure	11/09/12	BAL	na	na	Renal transplant	na	na	na	16
232	BE	Pure	29/11/12	BAL	F	65	Renal transplant	Belgium	First	No	16
236	BE	Pure	17/08/13	BAL	F	47	Renal transplant	Belgium	First	No	16
238	BE	Pure	13/12/13	BAL	M	59	Renal transplant	Belgium	First	No	16
239	BE	Mixed	01/07/13	BAL	M	64	Haematology	Belgium	First	No	16
243	BE	Pure	07/05/14	BAL	F	69	Renal transplant	Belgium	First	No	16

BAL: Bronchoalveolar lavage; BA, Bronchial aspirate, IS: immunosuppression; na: not available.

### 3.4 Temporal Distribution of the Genotypes

**Fig. 5:**
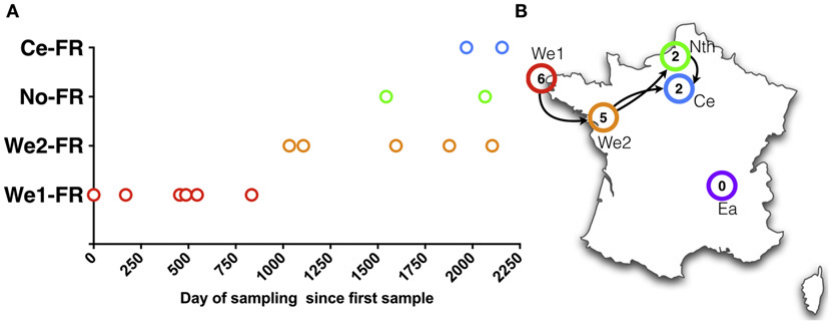
Temporal and geographical distribution of Gt123 in France. The number of days between the first sample and the corresponding sample harbouring Gt123 is reported on the X axis (Panel A). Most probable hypothesis regarding the temporal and geographical circulation of Gt123 in France (Panel B). The number of cases in the corresponding centre is indicated in the circles. Each centre is coloured adequately as red for We1-FR; orange for We2-FR; green for Nth-FR and blue for Ce-FR and purple for Ea-FR. (For interpretation of the references to colour in this figure legend, the reader is referred to the web version of this article.)

Gt123 has been isolated from 2009 to 2015, first in We1-FR (2009–2011) and then in other French centres (We2-FR, No-FR and Ce-FR) between 2012 and 2015 (Table 1, Fig. 5). The German Gt123 was sampled in January 2013. Two 30–40 year-old patients harbouring pure Gt123 were born in Caribbean islands and in Algeria before kidney transplantation was performed in Ce-FR in 2014 and 2015, respectively.

We found only two other genotypes that were recovered > 6 times between 2012 and 2015. In comparison, Gt16 (n = 6) was recovered two times in 2012, three in 2013 and one in 2014 and not in 2015. Gt94 was not recovered in 2012 and 2013 and recovered four times in 2014 and twice in 2016.

## 4 Discussion

The present *P. jirovecii* genotyping study is the first one dealing with several European countries and using an MLP typing (Gits-Muselli et al., 2015). In analysing 249 cases of PCP recruited from 16 centres across 12 European countries, our main findings were the large proportion of PCP cases harbouring mixtures of FIs, the limited genetic evolution of *P. jirovecii* across Europe, and the possible enrichment of genotypes at some centres, possibly linked to the underlying disease of the patient.

The association between underlying disease/conditions and mixed infection has never been reported. We observed a significant increase of samples containing multiple FIs in HIV patients and a significant decrease of samples containing multiple FIs in renal transplant patients. This could be explained by the evolution of HIV infection over several years with a progressive decrease of about 61 CD4 T-cells/μL per year (Wolbers et al., 2010), that could allow the HIV-positive patient to inhale numerous and various *P. jirovecii* genotypes over the course of HIV infection. Then, PCP would occur with a mixture of genotypes encountered by the patient over the last 5–10 years. Conversely, renal transplant recipients have a controlled period of immunosuppression, resulting in a shorter duration for exposure to *P. jirovecii*, during which *P. jirovecii* would be able to proliferate rapidly, resulting in acute disease (124 to 170 days) (Phipps et al., 2011), the starting point potentially being the stop of anti-PCP prophylaxis (de Boer et al., 2011). Similar results were recently reported with a dominant genotype responsible for an outbreak in a haematology ward suggesting a recent acquisition of the epidemic genotype (Robin et al., 2017). Since PCP occurs when prophylaxis is stopped (de Boer et al., 2011), this raises the question of life-long administration of anti-PCP prophylaxis in at-risk patients, especially in renal transplant recipients with prolonged immunodepression (Alanio and Bretagne, 2017, Goto et al., 2017, Wang et al., 2012). However, the fungal load is known to be lower in HIV-negative patients (Alanio et al., 2011, Cordonnier et al., 2016, Roux et al., 2014) than in HIV-positive patients and a low fungal load could negatively influence the detection of mixed infections. Thus, only the most numerous would be evidenced whereas multiple genotypes would be nevertheless present. Using a single nucleotide extension method, which can detect minority alleles until 5–10%, the low fungal loads were not associated with less mixtures than the high fungal loads, suggesting an independent association between number of genotypes with underlying diseases rather than with the fungal load (Alanio et al., 2015). To avoid biases in the interpretation of the results, it was decided not to genotype PCP cases harbouring mixtures of isolates with more than one allele in more than one marker. To interpreted these mixtures, as proposed by other authors (Parobek et al., 2014), would have introduced genotypes which the reality was impossible to ascertain. In addition, in 31% of the samples it was not possible to obtain all makers. This was likely as a result of low DNA fungal loads, degradation of DNA upon storage or shipment, or DNA extraction issues. With these restrictions to the 69% of analysable samples, we found a global prevalence of mixture at 64% (160/249), in the same order of the 70% of mixtures previously reported (Alanio et al., 2016, Gits-Muselli et al., 2015, Hauser et al., 2001, Parobek et al., 2014). However, more sensitive methods such as ultra deep pyrosequencing have detected mixtures in up to 92% of the samples (Alanio et al., 2016). These results suggest, as already described (Hauser et al., 2001, Parobek et al., 2014), that PCP is frequently due to multiple genetically distinct organisms, that are unlikely to be diploid. Indeed, *P. jirovecii* is known to be a haploid organism (Cissé et al., 2012, Wyder et al., 1998), although lack of detailed genetic analyses prevents the detection of DNA insertion or deletion leading to aneuploidy. However, in the situation where multiple alleles at one locus are observed (n = 56), duplication of part of the genome, resulting in aneuploidy, could be responsible for this diversity. Microevolution of part of the *P. jirovecii* organisms could also explain this phenomenon.

Even if the DP of the method calculated from this study is > 0.99 (DP = 0.995, as compared to 0.992 in our previous study (Gits-Muselli et al., 2015)), the distance between *P. jirovecii* genotypes remains low using our MLP typing method, resulting in a limited number of genetic groups. This low genetic disparity had been suggested by Parobek et al. when they found that *P. jirovecii* samples from San Francisco (n = 49) and from Spain (n = 29) had limited genetic differentiation (Parobek et al., 2014), as compared with Ugandan samples. This limited genetic divergence of European *P. jirovecii* isolates could be explained by specific aspects of *P. jirovecii* biology. Indeed, *P. jirovecii* is considered as an obligate fungal biotroph harbouring parasitic behaviour (Cissé et al., 2014), that may intrinsically limit the opportunities to diverge genetically. Loss of genes is the main characteristics of *P. jirovecii* genome evolution compared to its last common ancestor *Taphrina deformans* (Cissé et al., 2014, Ma et al., 2016). The DNA polymerases or the DNA repair machinery of *P. jirovecii* could also specifically restrict genetic diversity.

Our study highlighted that transmission may occur in specific centres (We1-FR and Gt123, UK and Gt94; BE and Gt16) or within different centres of a given country (FR centres and Gt123). This observation suggests that a more systematic genotyping analysis of PCP cases could lead to identification of clustered cases and to the implementation of prevention procedure to stop circulation of a specific Gt at a specific place. To our knowledge, some centres (including the CHU Vaudois, Lausanne, Switzerland and Necker University Hospital, Paris, France) already use air prevention measures such as mask wearing when a case of PCP is diagnosed.

Interestingly, the percentage of samples harbouring a mixture of FIs at a given centre dropped from 70% in average to 36–42% (We1-FR and BE). This suggests nosocomial PCP as a result of recent exposure to a given *P. jirovecii* genotype. This also suggests that a given genotype could have a biological advantage over other genotypes resulting in an increased number of patients with PCP due to this genotype. This hypothesis could be reinforced by the fact that Gt123 was responsible for 15 cases of PCP across France that could be considered independent since no contact between these patients could be imagined (renal transplant patients, transplanted and always followed in separate centres). Interestingly, Gt123 was not observed in patients with other causes of immunosuppression, suggesting that Gt123 could not be considered as highly prevalent or currently circulating in France. Gt123 could be well adapted to renal transplant patient immunodeficiency and Gt123 could have spread from the West of France (We1-FR) from 2009 to 2011 to other centres (We2-FR then No-FR and Ce-FR) between 2012 and 2015 (Fig. 5). A similar hypothesis has already been suggested by Sassi et al. (Sassi et al., 2012), who described two distinct outbreaks of PCP in renal transplant recipients due to the same genotype in Zurich (Switzerland) and Munich (Germany) (Hauser et al., 2012, Sassi et al., 2012). These cities are located about 300 km apart, which is almost half the distance between the centres in We1-FR and Ce-FR (592 km). In addition, identical genotypes were detected in different countries, with a total of 21/134 (15.3%) samples harbouring the same genotype found in more than one country, with the founder genotype (Gt33) and the Gt56 found in ≥ 4 countries (from Seville, Spain to Cologne, Germany, 2230 km apart). This suggests that some genotypes could have disseminated across Europe, or that this typing method is not discriminative enough to observe differences between those isolates (Alanio et al., 2017). This observation and ours suggest that a given *P. jirovecii* genotype is able to circulate within an area of at least of 600 km (Hauser et al., 2012, Sassi et al., 2012). It is possible that some immunocompromised patients were exposed to this specific genotype through contact with immunocompetent carriers, to rather than direct contact between renal transplant recipients located in different centres.

When analysing the most frequent genotype (Gt123), the difference between the date of the first (28th May 2009) and the last (23th April 2015) observation was 2156 days (i.e., 5.9 years) (Fig. 5). If one hypothesises that interhuman transmission is the main mechanism in PCP, this is potentially the longest period of transmission of PCP ever reported and this finding raises the issue of the dissemination of a given genotype over time in different places. When studying a single hospital, the duration of transmission of a given genotype varied from 1 to 2 months to 31 months (2.6 years) (Yiannakis and Boswell, 2016) or 32.2 months (2.7 years) (Gits-Muselli et al., 2015). This possible long time of transmission may suggest that immunocompetent individuals, such as health care workers, could serve as a potential reservoir, since environmental reservoir is unlikely for this fungus (Alanio and Bretagne, 2017). Thus, two patients of the present study harbouring pure Gt123 were born and had lived > 30 years in Algeria and in Caribbean islands before kidney transplantation was performed in Paris, France. This Gt123 was probably acquired after the renal transplantation from recent exposure either by direct transmission or through a third source. Whatever the final mode of transmission, our finding argues against reactivation of resident *P. jirovecii* previously acquired in their country of origin. Another argument for the recent acquisition of a new genotype in PCP is our observation of some temporal relationships between the genotype and the date of PCP (for example, 2012–2013 and Gt16, or 2014–2015 and Gt94). However, a limit to be acknowledged to our study on timing of transmission is that the samples were obtained from patients managed from 1998 to 2015. In particular, the centres with a low number of cases included the oldest cases. This was inherent to the retrospective design of our study and the uneven sampling prevents any definitive conclusion about the temporal evolution of the genotypes circulating across Europe. To address this question, specific prospective studies should be implemented in various European places.

In conclusion, the genetic diversity of *P. jirovecii* appears limited, suggesting a constant exchange between human individuals and the simultaneous presence of several genotypes in a given individual. On this constant exchange background, some temporal and geographical evolutions of genotypes were observed in independent centres suggesting recent acquisition of new genotypes and more rapidly circulating genotypes between immunocompromised patients. This reinforces the supposed dynamic transmission of *P. jirovecii* between non-immunocompromised and immunocompromised hosts, the first being reservoir from which new genotypes can be contracted by immunocompromised patients and subsequently transmitted to other patients (Alanio and Bretagne, 2017).

The following are the supplementary data related to this article.

Supplementary Table 1 Clinical and microbiological characteristics of the all the 249 patients and samples including mixed amples. Supplementary Table 1

Supplementary Table 2 Clinical and microbiological characteristics of the samples harboring the 201s fungal individuals. Supplementary Table 2
